# Anti-tumoral potential of MDA19 in human osteosarcoma via suppressing PI3K/Akt/mTOR signaling pathway

**DOI:** 10.1042/BSR20181501

**Published:** 2018-12-14

**Authors:** Bin Liu, Liang Xu, E-Nuo Dai, Jia-Xin Tian, Jian-Min Li

**Affiliations:** 1 Departments of Traditional Chinese Medical Orthopedics, Affiliated Hospital of Shandong Academy of Medical Sciences, Ji’nan, Shandong 250031, P.R. China; 2 Department of Orthopedics, Qilu Hospital of Shandong University, Ji’nan, Shandong 250012, P.R. China; 3 Departments of Orthopedics, Affiliated Hospital of Shandong Academy of Medical Sciences, Ji’nan, Shandong 250031, P.R. China

**Keywords:** EMT, MDA19, Osteosarcoma, PI3K/Akt/mTOR signaling

## Abstract

Osteosarcoma (OS) is the most common primary malignancy of skeleton with higher mortality rates amongst children and young adults worldwide, whereas effective and secure therapies have also been sought by researches with ongoing efforts. The purpose of the present study was to investigate the impact of N′-[(3Z)-1-(1-hexyl)-2-oxo-1,2-dihydro-3H-indol-3-ylidene] benzohydrazide (MDA19) on OS and explore its potential mechanism. Cell Counting Kit-8 (CCK8) and colony formation assay were employed to evaluate the potential effect of MDA19 on U2OS and MG-63 cells proliferation. Moreover, transwell migration and invasion assay were performed to assess the influence of MDA19 on U2OS and MG-63 cells migration and invasion. In addition, Annexin V-FITC/propidium iodide (Annexin V-FITC/PI) staining and flow cytometry were used to examine apoptotic ratio of the U2OS and MG-63 cells. Meanwhile, Western blot analysis was applied to explore change of relevant mechanism proteins in OS cells treated with MDA19. Our study showed that MDA19 had anti-proliferative activity of OS cells in a dose- and time-dependent manner, simultaneously, inhibition of colony formation was also observed in U2OS and MG-63 cells after incubation of MDA19. Besides, MDA19 could significantly inhibit the number of migrated and invaded OS cells and markedly increase the OS cells apoptosis rate. Mechanistically, we detected detectable reductions in apoptosis related proteins, epithelial–mesenchymal transition (EMT)-related proteins and activity of phosphatidylinositol 3-kinase (PI3K)/Akt/mammalian target of rapamycin (mTOR) signaling in U2OS and MG-63 cells exposure to MDA19. Overall, the current study indicates *in vitro* anti-proliferative, anti-metastatic, and pro-apoptotic potential of MDA19 in U2OS and MG-63 cells. Our findings propose a clue for further studies with this compound in preclinical and clinical treatment for OS.

## Background

Osteosarcoma (OS), also termed as osteogenic sarcoma, is the most common malignant bone tumor worldwide, marked by malignant tumor cells directly form bone tissue and mainly harming children and young adults [1,2]. Standard therapies of OS encompass surgical operation, neoadjuvant and adjuvant chemotherapy. Surgical operation has resulted in amputations, additionally, neoadjuvant or adjuvant chemotherapy have induced toxic effect(s) unable to be prevented efficiently, albeit these strategies have improved survival rate of patients, while an overall 5-year survival rate is still disappointingly low (below 30%) in patients with relapse and metastasis [3,4]. Therefore, identification and validation of some pharmaceutical-targetted therapies against protein molecules or other biomarkers in OS still is imperative to treat OS for further clinical applications. Several pharmaceuticals have been recently investigated for OS such as liposomal MTP-PE, bisphosphonates, and so forth [5–7]. However, little report is recently available for the role of N′-[(3Z)-1-(1-hexyl)-2-oxo-1,2-dihydro-3H-indol-3-ylidene] benzohydrazide (MDA19) in anti-tumoral effect of OS.

It is well-known that MDA19 is a selective agonist at cannabinoid 1 receptor (CB_1_R) and CB_2_R, but this agonist effect is disparate in human and rat, exerting normal agonist action at human CB_1_R or CB_2_R and at the rat CB_1_R while as an inverse agonist at the rat CB_2_R [8]. It is reported that CBR activation could abate cancer progression [9,10], and thus CBR is utilized as a target for anti-carcinogenic therapy in multifarious types of cancer including human non-small lung cancer, glioma cancer, bladder cancer, breast cancer, prostate cancer, and so on [11–13]. Further, previous researches have reported that CBR selective agonists are capable of inducing potential anti-proliferative and apoptotic actions [14,15]. Additional studies have indicated that cancer cell invasion and metastasis are down-regulated following treatment with CB_2_ selective agonists [16,17]. It is well characterized that invasion and metastasis are closely associated with epithelial–mesenchymal transition (EMT) program [18]. A large body of evidence has manifested that bone cancer cell metastasis is a prominent feature in OS emergence [19]. Accumulating studies have illustrated that modulation of CBR enable to affect bone metastasis [20]. Nevertheless, there are rare researches associated with MDA19 on OS, particular in mechanism of action.

Based on above background, we hypothesize that MDA19 might have an anti-proliferative effect on OS cells. Thus, in the present study, we investigated the role of MDA19 in the probable inhibition of OS cells growth, invasion, migration, and induction of OS cells apoptosis, thereupon explored EMT and the molecular pathways phosphatidylinositol 3-kinase (PI3K)/Akt/mammalian target of rapamycin (mTOR) potentially mediating the anti-proliferative, anti-metastatic, and pro-apoptotic action of MDA19 on OS.

## Materials and methods

### Drugs and reagents

Telmisartan was purchased from MedChemExpress Biotechnology (Shanghai, China). Primary antibodies against the Akt (#9272), p-Akt Ser^473^ (#4060), mTOR (#2972), and p-mTOR (#2971), P70S6K (#9202) and p-P70S6K (#9208) were purchased from Cell Signaling Technology, Inc. (Danvers, MA, U.S.A.). Primary antibodies against the vascular endothelial growth factor (VEGF) (ab53465), β-Catenin (ab32572), N-Cadherin (ab18203), E-Cadherin (ab1416), Slug (ab106077), Twist (ab49254) were purchased from Abcam (Cambridge, U.K.). Primary antibodies against the Bcl-2 (Cat. No. 12789-1-AP), Bax (Cat. No. 50599-2-Ig), Cleaved Caspase-3 (Cat. No. 19677-1-AP), Cyclin D1 (Cat. No. 60186-1-Ig), Gapdh (Cat. No. 60004-1-Ig), and peroxidase conjugated secondary antibodies (goat anti-mouse: SA00001-1, goat anti-rabbit: SA00001-2) were obtained from Proteintech Group, Inc (Wuhan, China). The ECL detection system was from Proteintech Group, Inc (Wuhan, China).

### Cell culture

Human OS cell lines U2OS and MG-63 were obtained from American Type Culture Collection (Manassas, VA, U.S.A.). Cells were routinely cultivated in Dulbecco’s modified Eagle’s medium (DMEM) (Gibco Invitrogen Corporation, NY, U.S.A.) supplemented with 10% FBS, containing penicillin (100 U/ml) and streptomycin (100 μg/ml) at 37°C in a 5% CO_2_ humidified atmosphere. Cells in exponential growth phase (approximately 1 × 10^6^ cells/ml) were used for the following experiment.

### Cell proliferation assay

Proliferation of cells was determined by Cell Counting Kit-8 (CCK-8) according to the manufacturer’s protocol. Approximately 5 × 10^3^ cells were plated in a 96-well plate. After overnight culture, cells were administrated with MDA19 (0–200 μM) for 72 h. At 72 h following MDA19 administration, medium was discarded and then CCK-8 reagent (10 μl) was added to each well, following which the plates were incubated at 37°C for 1.5 h. Absorbance value (optical density, OD) was measured at 450 nm using a microplate reader. According to the effect of concentration gradient, the drug concentration of the following experiment was determined. Experimental groups cells with MDA19 at this concentration and negative control groups (NC) cells with 1% DMSO in culture media were cultured for 24, 48, and 72 h, respectively. Cell Counting Kit-8 (CCK8) detection procedures were similar as above.

### Plate clone formation assay

The anti-proliferative activity of MDA19 on U20S and MG-63 cells was assessed by colony-forming assay. Briefly, 300 cells were seeded into 6-cm plates containing 5 ml culture medium and gently rotated to allow the cells to disperse evenly for 48 h. Afterward, cells were treated with MDA19 and cultured for 7–14 days changing fresh medium every 3 days. Then, cells were observed frequently and terminated off culture when there was a visible clone in culture vessel, subsequently, supernatants were removed, cells were washed with PBS, fixed with 4% paraformaldehyde for 30 min, and stained with 0.1% Crystal Violet for 30 min. After washing with water slowly, the plates were air dried. The total number of colonies was counted directly by naked eye.

### Migration and invasion assays

Migration and invasion assays were executed using transwell chambers (Corning Costar, Cambridge, MA, U.S.A.) with membrane pore size of 8.0 μm (BD Biosciences, San Jose, CA) and without/with matrigel (BD Biosciences, San Jose, CA) according to the manufacturer’s protocol. Approximately 1 × 10^5^ cells were suspended in 100 μl serum-free culture medium and plated in the upper chamber, whereas the lower chamber was suffused culture medium with 10% FBS. After incubation for 24 h at 37°C, 5% CO_2_, non-migrating cells on the top chamber were scraped off using cotton-tipped swabs, and then cells migrating through the membrane were fixed with 4% paraformaldehyde for 30 min and stained with 0.1% Crystal Violet for 20 min. The migrated cells in the bottom of the chamber were counted under a microscope.

For cell invasion detection, the steps were similar to detection of cells migration, but matrigel was plated in the transwell inserts.

### Flow cytometry assay of apoptosis

For apoptosis analysis, (Annexin V-FITC) Annexin V-FITC/propidium iodide (PI) staining was performed by flow cytometry using an Annexin V-FITC apoptosis detection kit (Beijing 4A Biotech, Beijing, China) according to the manufacturer’s directions. After 48 h of MDA19 treatment, the cells were washed twice with cold PBS, incubated with Annexin V-FITC/PI for 5 min at room temperature in the dark. The apoptotic cells were analyzed with FACScan flow cytometry (FACS Calibur, BD Biosciences, CA).

### Western blot analysis

For Western blot analysis, the proteins were collected using RIPA lysis buffer (CWBIO, Beijing, China). Protein concentrations were measured using a BCA Protein Assay Kit (CWBIO, Beijing, China). Equal amounts of protein were separated by 8–12% Tris-glycine gradient gels via SDS/PAGE and transferred on to PVDF membranes. The membranes were blocked with 5% non-fat milk in TBS buffer containing 0.1% Tween-20 and then incubated with primary antibodies (1:1000 dilution) against AKT, p-AKT, mTOR, p-mTOR, Cyclin D1, P70S6K, p-P70S6K, VEGF, β-Catenin, N-Cadherin, E-Cadherin, Slug, Twist and Bcl-2, Bax, Cleaved Caspase-3, and against Tubulin (1:5000 dilution) at 4°C overnight, followed by horseradish peroxidase–conjugated secondary antibodies (1:5000 dilution). Western blot bands were visualized by ECL kit and were measured with QUANTITY ONE software.

### Statistical analysis

Statistical analyses were performed using the SPSS 22.0 software (International Business Machines Corporation, New York, U.S.A.). Data were processed as mean ± S.D. The differences were analyzed by the Student’s *t* test and a one-way ANOVA. For one-way ANOVA, post hoc test was performed using Dunnett’s *t* test. The differences were considered to be statistically significant between two groups if *P<0.05.

## Results

### Effect of MDA19 on proliferation of OS cells

**Figure 1:**
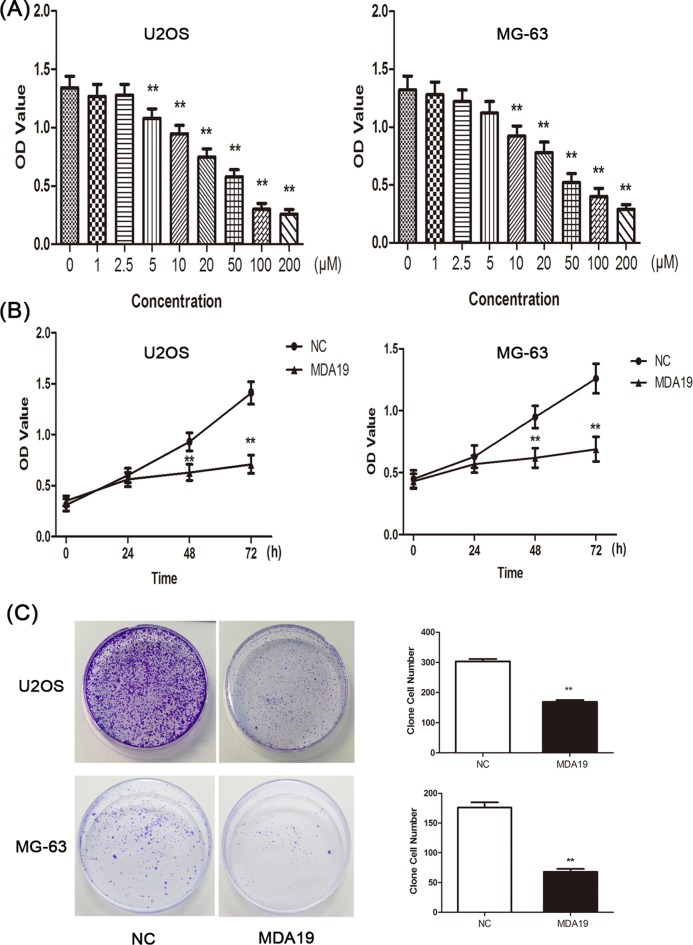
The potential activity of MDA19 on OS cells proliferation (**A**) Effect of MDA19 on U2OS and MG-63 cells’ viability after incubation at MDA19 concentrations 0–200 µM was detected by CCK8 assay. (**B**) Effect of MDA19 on U2OS and MG-63 cells’ viability after incubation at 0–72 h of MDA19. (**C**) Colony formation test was executed to examine U2OS and MG-63 cells proliferation after treatment with MDA19. Values are presented as mean ± S.D. **P<0.01 compared with NC groups.

To investigate whether MDA19 could affect the proliferation of OS cells, CCK8 assay and colony formation assay were performed. First of all, we determined cells’ viability by measuring OD value after U2OS and MG-63 cells treated with MDA19 at 0, 1, 2.5, 5, 10, 20, 50, 100, 200 µM and examined that the inhibitory effect of MDA19 on OS cells’ viability was in a concentration-dependent manner. However, at concentration higher than 20 µM, the OS cells’ viability was lower than 50% compared with that of 0 µM (Figure 1A), which was improper for the following experiments. To facilitate the study, we chose 20 µM as a valid concentration in the next experiments. Moreover, we observed that prohibitive action on U2OS and MG-63 cells by treated with MDA19 was in a strongly time-dependent mode (Figure 1B). Simultaneously, for detection of OS cells proliferation by colony formation assay, we found that number of colonies of U2OS and MG-63 cells were markedly reduced in MDA19 groups comparable with NC groups (Figure 1C, F (8, 18) = 85.396, Figure 1C, F (8, 18) = 52.296, **P<0.01). In short, these results showed that the proliferation abilities of U2OS and MG-63 decreased evidently after treatment with MDA19.

### Effect of MDA19 on migration and invasion of OS cells

**Figure 2:**
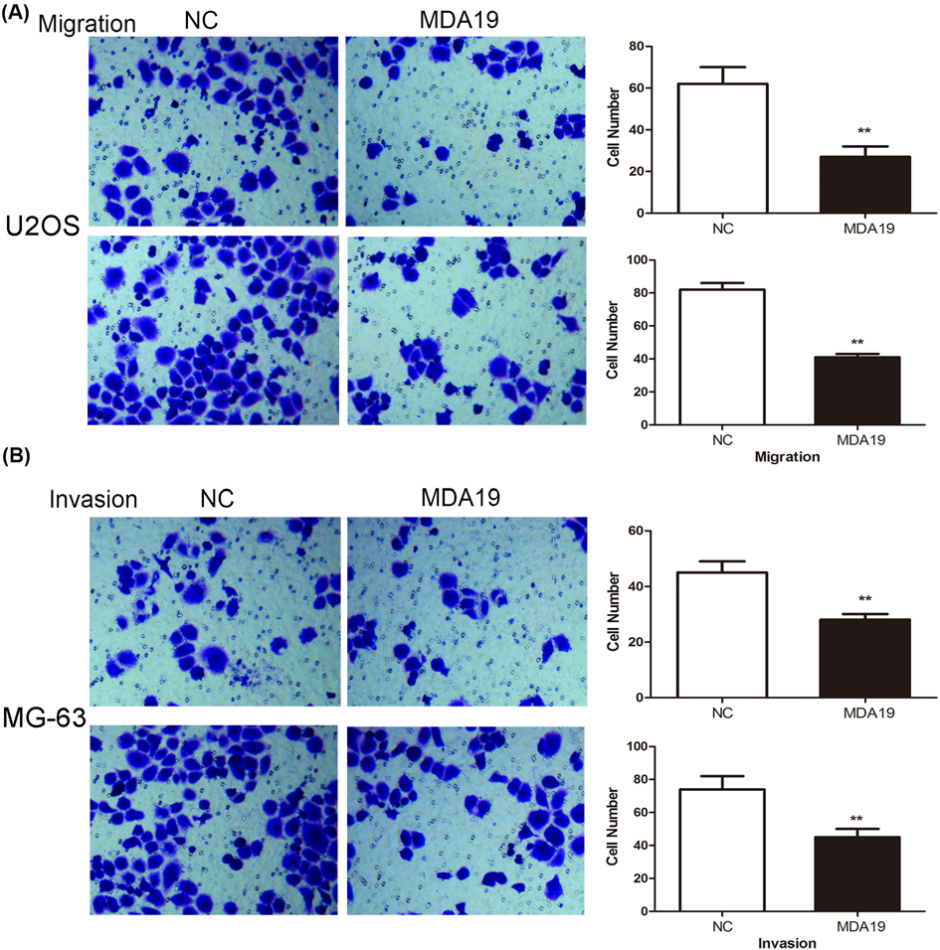
The impact of invasive and migratory capacities in OS cells treated with MDA19 was detected by transwell invasion and migration assay (**A**) The change of invaded U2OS and MG-63 cells number after incubation of MDA19. (**B**) The change of migrated U2OS and MG-63 cells number after incubation of MDA19. All data are presented as mean ± S.D. **P<0.01 compared with NC groups.

To study the potential effect of MDA19 on OS cells migration and invasion, transwell migration and invasion were performed. Figure 2A showed that the numbers of migrated cells after MDA19 treatment were significantly reduced compared with the NC groups (**P<0.01). Similarly, consequence of transwell invasion assay showed that MDA19 treatment obviously debased the number of invaded cells compared with NC groups (Figure 2B, **P<0.01).

### Influence of MDA19 on apoptosis in OS cells

**Figure 3:**
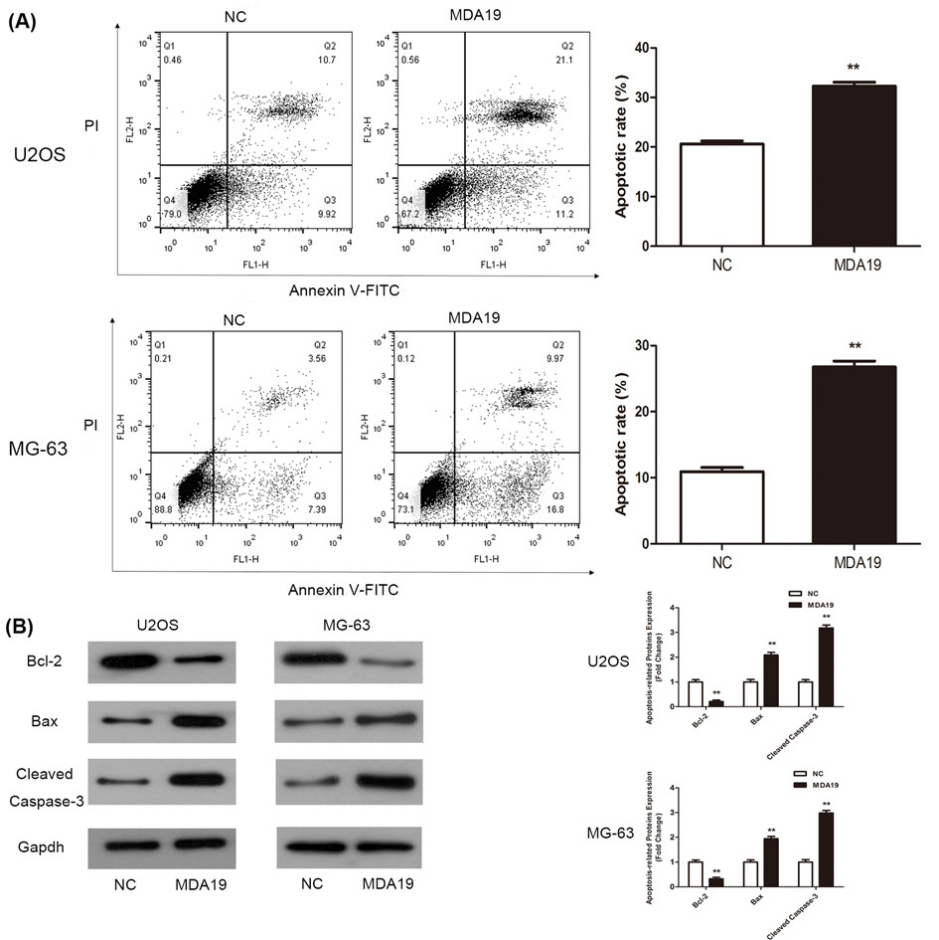
The effect of apoptosis in OS cells administrated with MDA19 was tested by flow cytometry after Annexin V-FITC/PI staining and Western blot assay (**A**) The change of apoptotic rate in U2OS and MG-63 cells after incubation of MDA19. (**B**) Western blot analysis was performed to test a series of antibodies (Bcl-2, Bax, Cleaved Caspase-3, and Gapdh). All values are expressed as mean ± S.D. **P<0.01 compared with NC groups.

To examine whether MDA19 influence OS cells apoptosis, flow cytometry assay with staining Annexin-V-FITC/PI was applied to test U2OS and MG-63 cells apoptotic ratio. Results discovered that cells apoptotic rate in MDA19 groups (32.3 ± 0.8%) was significantly decreased compared with NC groups (20.6 ± 0.6%) (Figure 3A, **P<0.01). Next, to investigate the molecular mechanism of apoptosis, we examined proteins closely related with apoptosis using Western blot assay. Western blot results revealed that after treating with MDA19, the expression level of anti-apoptotic protein Bcl-2 was reduced while that of pro-apoptotic protein Bax was increased markedly compared with NC groups (Figure 3B, **P<0.01). In addition, the expression level of Cleaved Caspase-3 was increased significantly comparable with NC groups (Figure 3B, **P<0.01). Briefly, above results indicated that MDA19 could induce OS cells apoptosis effectively.

### The potency of MDA19 on change of EMT proteins in OS cells

**Figure 4:**
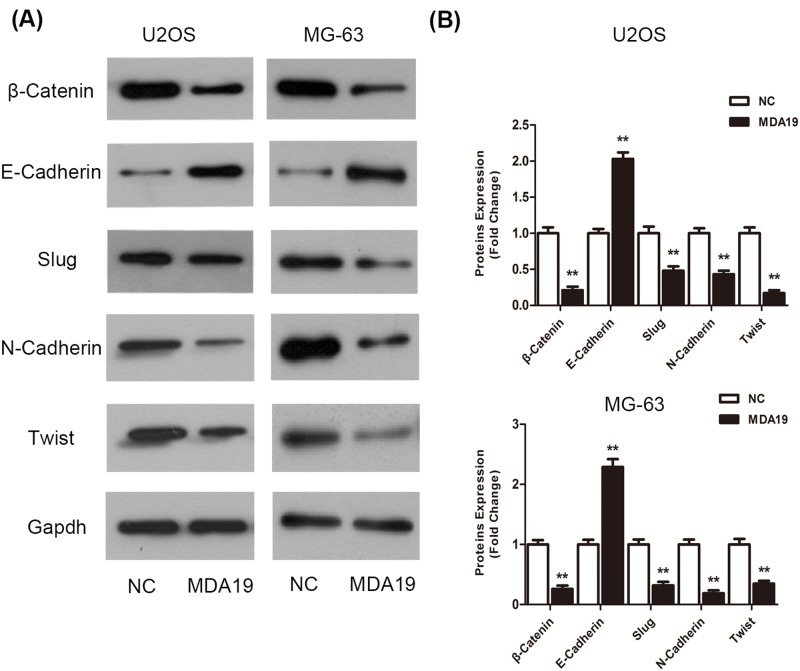
The potency of EMT in OS cells treated with MDA19 was tested by Western blot assay (**A**) Western blots analyze changes of EMT-related proteins in OS cells and proteins bands were quantitated. (**B**) Densitometric quantitation of protein bands were measured by QUANTITY ONE software. All values are expressed as mean ± S.D. **P<0.01 compared with NC groups.

It is reported that EMT is regarded as a crucial representation in cancer metastasis and invasion and proved to promote cell growth and survival [21,22]. Thus, to address molecular mechanism of MDA19 induced anti-metastatic effect on OS cells, we detected proteins closely related with EMT occurrence, such as the epithelial marker E-cadherin, correspondingly, the mesenchymal markers β-Catenin, N-cadherin, Slug, and Twist. Western blot assay results showed that E-cadherin was up-regulated evidently while β-Catenin, N-cadherin, Slug, and Twist were down-regulated markedly in MDA19 groups comparable with NC groups (Figure 4, **P<0.01 respectively).

### The impact of MDA19 on PI3K/Akt/mTOR signaling

Given that PI3K/Akt/mTOR has a crucial part in tumorigenesis and progression [23,24]. Importantly, several researches have claimed that EMT induction is concomitant with activation of the PI3K/Akt/mTOR pathway [25,26]. Thereby, we further investigated whether the key signaling participated in EMT mediating MDA19 potent inhibitory impact on OS cells proliferation and metastasis.

**Figure 5:**
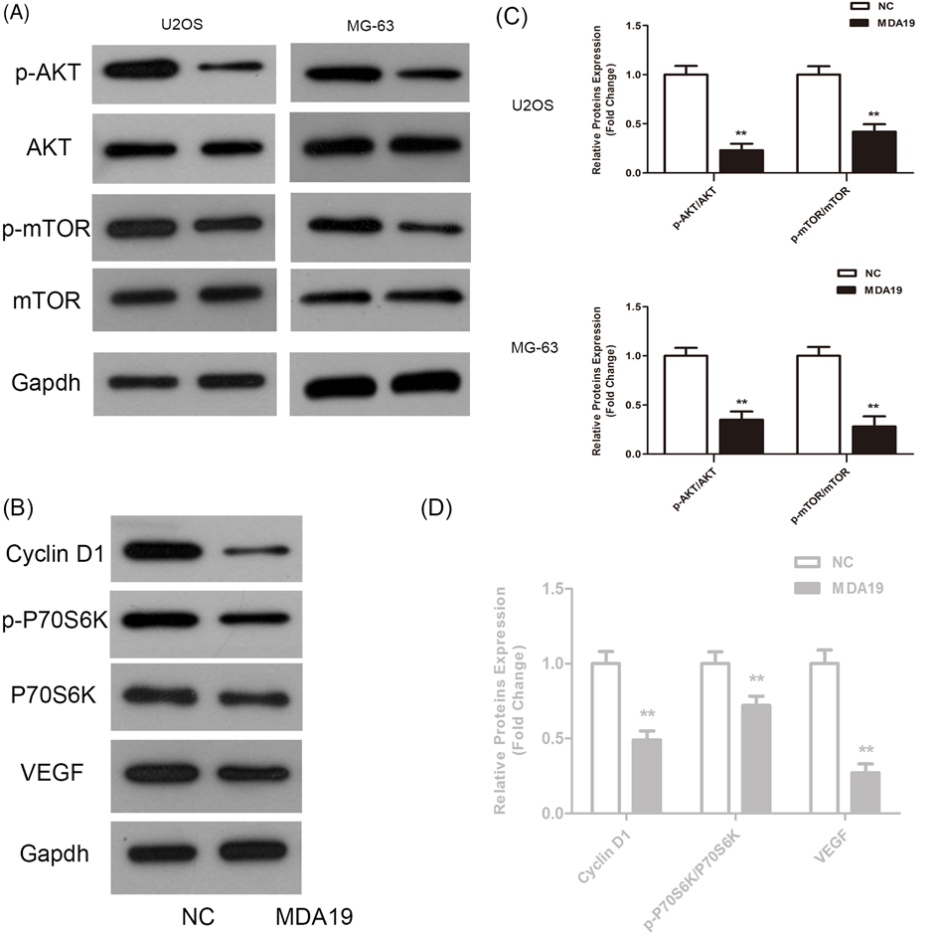
The activity of PI3K/Akt pathway was examined in OS cells exposure to MDA19 (**A and C**) Western blots analyze changes of PI3K/Akt pathway-related proteins in OS cells and proteins bands were quantitated. (**B and D**) Densitometric quantitation of protein bands were measured by QUANTITY ONE software. All values are expressed as mean ± S.D. **P<0.01 compared with NC groups.

We detected several key molecules relevant with this crucial pathway by Western blot assay. The results showed that administration of U2OS cells with MDA19 results in dramatic decrease in phosphorylation levels of Akt and mTOR, while no obvious changes were observed in the total Akt and mTOR level. Moreover, downstream factors of the key signaling like Cyclin D1, P70S6K, and p-P70S6K related to cell proliferation showed an evident down-regulation in MDA19 groups compared with NC groups (Figure 5, **P<0.01). Further, based on previous evident that VEGF, upstream factor of the key signaling and the most important pro-angiogenesis factor, has the potential to promote cell growth and metastasis, we examined the VEGF expression and found a notable reduction in it in MDA19 groups in comparison with NC groups (Figure 5, **P<0.01).

## Discussion

In the present study, we functionally demonstrated the potential role of MDA19 on cell proliferation, migration, invasiveness, and apoptosis in OS cells. Additionally, we observed a detectable change in EMT proteins and PI3K/Akt/mTOR signaling following OS cells exposure of MDA19, which implies that MDA19 might be a potential therapeutic agent against OS in the future clinical practice.

It has been demonstrated that MDA19, as a CBR selective agonist, has a potential of alleviating neuropathic pain [8]. Nevertheless, with incidence of various cancers and advancement of abundant research, CBR selective agonist was considered as an anti-tumor agent in several cancers [27–29]. Emerging specific reports have epitomized that selective CBR agonists are able to exert anti-carcinogenic activities by affecting cell survival, cell proliferation, or cell death in several types of cancers [30,31]. Another report has depicted that CBR deficiency could restrain loss of bone [32,36]. Growing data have disclosed that targetting CB_2_R in the bone possesses potential efficacy in the reduction in bone syndrome related to cancer [33]. In spite of this, there are no data available to date concerning the function of MDA19 on OS. Herewith, in the present study, MDA19 was found to potentiate anti-proliferative and apoptotic actions in OS cells.

OS is one of the most common malignant bone cancers in children and adolescents, featured by rapid cancer cell growth and high metastasis. EMT is a critical step for the transformation of early-phase tumors into invasive malignancies and regarded as closely correlated with cancer invasion and migration [34,35]. Characteristic reduction in E-cadherin is considered as the pivotal manifestation of EMT. Beyond that, Slug and Twist, prominent transcriptional suppressors of E-cadherin, are capable of binding to specific domain of the E-cadherin promoter [36]. It is known that N-Cadherin as mesenchymal marker plays a crucial role in inducing EMT process. Additionally, β-catenin is known to be one of the hallmarks for the initiation of the EMT [37]. In the light of these theories and data, we examined these tightly relevant proteins and found that Western blotting results were in-line with above evidence. Collectively, all results indicated that EMT mediated MDA19 acting on anti-proliferative and anti-metastatic characteristics in OS cells, however, therein underlying molecular mechanism of action was unclear yet.

It is well known that VEGF is regarded to be the key factor to facilitate angiogenesis involved in the whole process of OS growth, invasion, and migration [38,39]. Meanwhile, VEGF has been reported to be an upstream regulator of PI3K/Akt/mTOR signaling [40,41]. Further, activation of CBR in cancer cells has been reported to be relative to obvious prohibition of tumor angiogenesis resulting from the down-regulation of tumour-derived VEGF [42]. In-line with these evidence, we examined VEGF was down-regulated in MDA19 inhibiting OS cells growth, invasion, and migration.

Aberrant activation of PI3K/Akt/mTOR is recognized to play a pivotal role in modulating cell proliferation and apoptosis especially in tumor progress [23,24]. Thus, targetting this signaling by some chemicals, namely hexamethylene bisacetamide [43], Y-tocotrienol [44], DAW22 [45], and Ginsenoside Rg5 [46], is explored to be a therapeutic strategy for treating cancers. Several studies have reported that EMT program is accompanied by activation of PI3K/Akt/mTOR [25,26]. Additional study has shown that Akt is able to induce EMT [47]. Consistent with these results, our findings revealed that key factors of the signaling, p-Akt and p-mTOR were decreased after administration with MDA19, downstream factors like Cyclin D1, P70S6K, and p-P70S6K were also reduced by MDA19 treatment. In the meantime, the change of VEGF as an upstream factor was similar to that of above proteins. Some of effective mTOR inhibitors, such as sirolimus and everolimus, have been applied in animal model and even phase II trials, however, the prespecified target of 6-month progression-free survival (PFS) of 50% or greater has not been attained [48]. Besides this, the safety of them would need better management according to suggestions from some researchers [49]. Thus, combination of multidrug strategy has been proposed to apply in treating OS, which are more efficient than one of them only [50,51], although there are some of side effects unavoidably. Our current work could provide a potent contribution to further palliative care or neoadjuvant/adjuvant of OS. Altogether, above data indicated that MDA19 exerted potent prohibitory activity against OS cell proliferation, invasion, metastasis, and elicited EMT occurrence might via mediating inactivation of PI3K/Akt/mTOR pathway. Despite these data exhibited, there are some limits, namely, *in vivo* experiments needed to be carried out, based on present results with anti-cancerous function, in-depth mechanisms remains to be explored. Interestingly, we also focus on mTOR inhibitors and MDA19 comparison and even combination on effect of OS tumor properties in the further work.

In summary, the current study elucidated that MDA19 impeded efficiently cell proliferation, migration, and invasion as well as induced apoptosis in OS cells. Furthermore, we found MDA19 down-regulated key proteins for EMT process, mechanistically, PI3K/Akt/mTOR mediated MDA19 developing prohibitory effect of EMT process, which provided new insights into the MDA19 potential anti-tumoral action and relevant molecular mechanisms in OS. Collective findings support a clue that MDA19 might be an alternatively novel candidate for OS therapy.

## Abbreviations

CB_1_R: cannabinoid 1 receptor
CCK8: cell counting kit-8
EMT: epithelial–mesenchymal transition
Gapdh: Glyceraldehyde-3-Phosphate Dehydrogenase
MDA19: N′-[(3Z)-1-(1-hexyl)-2-oxo-1,2-dihydro-3H-indol-3-ylidene] benzohydrazide
mTOR: mammalian target of rapamycin
MTP-PE: muramyl tripeptide phosphatidyl ethanolamine
NC: negative control
OD: optical density
OS: osteosarcoma
PI: propidium iodide
PI3K: phosphatidylinositol 3-kinase
P70S6K: Ribosomal Protein S6 Kinase B1
RIPA: Radio Immunoprecipitation
VEGF: vascular endothelial growth factor
